# Knowledge, attitude, and proficiency of healthcare providers in cardiopulmonary resuscitation in a public primary healthcare setting in Qatar

**DOI:** 10.3389/fcvm.2023.1207918

**Published:** 2023-07-18

**Authors:** Shajitha Thekke Veettil, Mohamed Shaheen Anodiyil, Hanan Khudadad, Mohamed Ali Kalathingal, Abdul Hakeem Hamza, Femina Purakaloth Ummer, Ahmed Sameer Alnuaimi

**Affiliations:** ^1^ Clinical Research Department, Primary Health Care Corporation, Doha, Qatar; ^2^ Al Rayyan Health Center, Primary Health Care Corporation, Doha, Qatar; ^3^ Al Daayen HC, Primary Health Care Corporation, Doha, Qatar; ^4^ Umm Ghuwailina HC, Primary Health Care Corporation, Doha, Qatar; ^5^ Al Mashaf Health Center, Primary Health Care Corporation, Doha, Qatar

**Keywords:** cardiopulmonary resuscitation (CPR), knowledge, skills, healthcare providers, primary care, Qatar

## Abstract

**Introduction:**

Early and effective cardiopulmonary resuscitation (CPR) increases both survival rate and post–cardiac arrest quality of life. This study aims to assess the current knowledge and ability of physicians and nurses in health centers (HCs) operated by the Primary Health Care Corporation (PHCC) in Doha, Qatar, to perform CPR.

**Methodology:**

This study consists of two parts. The first part is a descriptive cross-sectional survey using an online form targeting physicians and nurses working in all HCs to assess their CPR knowledge and attitude. The second part is a direct observation of CPR drills to evaluate the skills and competencies of code blue team members in a convenient sample of 14 HCs. A multivariate model was employed to test the independent effect of explanatory variables on the total knowledge score.

**Results:**

A total of 569 physicians and nurses responded to the survey. Only one-half (48.7%) formally received training on basic life support within the last year. Furthermore, 62.7% have tried to revive a dying person with no pulse. All the participants recognize the importance of knowing how to revive a dying adult or child as part of their job. The study showed that being a nurse was the most important predictor of a higher knowledge score in both components. Attending more resuscitation courses (3–6 courses in the last 3 years) ranked second in importance, and a longer experience in clinical practice (5–10 and >10 years) ranked third in predictive power. In addition, the direct observation of CPR drill performance revealed a satisfactory outcome.

**Conclusion:**

The level of CPR knowledge and skills practice among healthcare providers in PHCC is deemed satisfactory as most providers reported having performed CPR in the past. Considering that PHCC is the first step to people’s healthcare in Qatar, clinical staff should be certified and assessed regularly to ensure retention of resuscitation knowledge and skills.

## 1. Introduction

Early and effective cardiopulmonary resuscitation (CPR) increases the survival rate and post–cardiac arrest quality of life. In Qatar, there is a need for more data on the basic knowledge of CPR among healthcare providers (HCPs). Primary Health Care Corporation (PHCC) has a responsibility to deliver high-quality resuscitation service and to ensure that its staff is appropriately trained and regularly updated to a level of proficiency appropriate to each individual's expected role as defined in the current PHCC CPR policy and procedure in health centers (1). This study was designed to gather primary baseline data to assess CPR training needs for primary healthcare professionals in Qatar.

Recognition and early intervention in incidents of cardiac arrest save lives (2). Every minute without cardiopulmonary resuscitation (CPR) and defibrillation reduces the victim's chance of survival from cardiac arrest by 7%–10% (3). CPR is now modified into a simple version of skills anyone can learn regardless of formal medical training (4). This approach allows any trained staff member to quickly initiate this life-saving treatment in both hospitals and primary care settings (5).

In developed countries, the incidence of in-hospital cardiac arrest attended by a resuscitation team and receiving CPR is estimated to be approximately 2 per 1,000 admissions (6). In such settings, training of staff members, the introduction of resuscitation teams, and pre-employment requirements of CPR certification have resulted in improvements in outcomes of in-hospital cardiac arrest (7). However, in developing countries, available data on the incidence, organization, and outcome of in-hospital facility-based CPR are limited (8).

The American Heart Association (AHA) standards have been adopted in resuscitation protocols in Qatar. Each PHCC-operated health center assigns a group of specifically trained clinical staff to code blue calls. The code blue team (CBT), consisting of two physicians and four nurses, provides immediate response and management of care to a patient who has suffered cardiac and/or respiratory arrest until an ambulance arrives.

To ensure the delivery of high-quality CPR, drills/simulations are regularly conducted for members of the cardiac resuscitation team. The lead physicians and head nurses are responsible for arranging these drills. In PHCC, it is recommended that all the CBT members be certified in one or more of the following courses: Basic Life Support (BLS)-AHA/Advanced Cardiac Life Support (ACLS)-AHA (for adults) and Pediatric Life Support (PLS)-AHA/Pediatric Advanced Life Support (PALS-AHA) (1, 9). In addition, the Qatar Council for Health Practitioners (QCHP) requires the certification for license renewal (10).

In PHCC health centers (HCs), the physician-in-charge and the head nurse are responsible for ensuring that all members of the CBT are well-trained, competent, and adequately equipped for resuscitation cases. The competency levels of CBT are regularly reviewed, and their continuing training needs are identified (11).

The knowledge and ability of HCPs in performing CPR during the clinical practice were inadequately studied in Qatar. Therefore, it is important to evaluate these aspects to understand training needs better and initiate training and awareness programs according to those needs among primary healthcare providers (12). Understanding the current state of CPR knowledge and skills among these clinicians will help guide future training efforts and the implementation of strategies that will benefit healthcare delivery to patients with cardiopulmonary arrest.

## 2. Aim

This study aims to determine the current level of CPR knowledge and proficiency among physicians and nurses in primary care health centers in Doha, Qatar.

The primary objectives include the following:

• Measure the level of knowledge of health center physicians and nurses on CPR.
• Measure the level of CPR proficiency and skill of health center physicians and nurses.
• Assess the association between explanatory factors selected (such as gender, duration of clinical experience, professional affiliation, frequency of attending courses or practicing resuscitation, and being exposed to a real-life situation trying to revive/resuscitate a dying person/adult with no pulse) and the CPR proficiency/skill level of the primary care physicians and nurses.

## 3. Methodology

### 3.1. Study design

The study took place between the years 2020–2021. It is a cross-sectional study with two parts. The first part was a descriptive cross-sectional online survey of healthcare providers in all PHCC HCs to assess their CPR knowledge and attitudes. The second part relied on direct observation of study participants during CPR drills to assess the skills and competencies of a randomly selected sample of CBT members in all the health centers operated by PHCC, Qatar.

### 3.2. Study setting and population

PHCC is the main healthcare provider for the people of Qatar. At the time of the study, Primary Health Care Corporation was operating 27 primary healthcare centers distributed in three regions of Qatar, namely, central, western, and northern. A total of thirteen of these HCs are located in Doha city (central region), while the other centers are located in the northern and western parts of the country. The HCs provide appropriate and effective healthcare services focused on the needs of patients. The services delivered emphasize health promotion, prevention, diagnosis, treatment, and provision of long-term and appropriate support to patients and their families.

### 3.3. Target population

The target population of the study includes nurses and physicians from all 27 health centers, with a total of 724 physicians and 1,772 nurses. All the healthcare providers included in the study are licensed by the QCHP and practicing in health centers during the study period.

### 3.4. Sampling and sample size determination

In the first phase of the study (assessment of knowledge and attitude), an online survey targeting all the eligible population was conducted. Prospective participants were invited via email to participate in the study. A total of 569 physicians and nurses responded to the survey. In the second phase of the study, which involved direct observation of CBT members in a CPR drill, only 14 out of the 27 HCs participated because of restrictions associated with the COVID-19 pandemic. All six CBT members in each health center were invited to participate. Each CBT includes six members, with two physicians and four nurses.

### 3.5. Data collection

A structured self-administered online questionnaire was utilized to conduct the cross-sectional study. The questionnaire items have previously been tested for validity and reliability in previous studies (11, 13). It covered several topics, including basic demographics, clinical experience, level of training, work site, BLS/CPR certification, and frequency of BLS/CPR courses.

The demographic part of the questionnaire collected info regarding the participants’ age, sex, field of specialty, total years of clinical experience, and years of clinical experience with PHCC. The baseline CPR training and CPR experience questions included six items about the performance of CPR and prior CPR training completed in the last 3 years. The third part of the questionnaire consisted of 34 questions related to service experience (Supplementary Material S1).

After obtaining approval, the survey link was shared with the targeted population via email. The communication sent included survey-relevant information and instructions, which complied with PHCC research requirements. The survey questions were written in English since all nurses and physicians undergo CPR courses in the English language. The data collected were saved in a password-protected file, with access limited to authorized investigators (Supplementary File S1).

A sample of CBT members from all 27 health centers was contacted to assess CPR skills. The study participants signed informed consent for direct observation of routine drills, which were performed in the health center according to the institutional policy). A structured evaluation form was used to rate staff performance during the drill, including the initial approach and compression—ventilation techniques based on the American Heart Association practical skills testing benchmark. The questionnaire used in the study has been adapted and modified to suit local needs and resource availability (Supplementary Material S2). The drill observation checklist was tested on a small group of participants prior to implementation. The individual scores from the drill observation were assessed independently and in combination to produce an overall score for each participant. No further follow-up was required from the participants upon completion of the online questionnaire and/or skills demonstration checklist.

### 3.6. Knowledge score

The knowledge score was calculated by adding up the scores from 23 items. For the first 22 items, one point was given if the respondent correctly identified the following:

• The answer (there is no pulse, and the patient is not breathing) to the question of when to start CPR
• The optimum compression–ventilation ratio for children getting CPR when done by one person
• The need for healthcare providers to minimize interruptions in chest compressions to less than 10 s
• The first action taken when finding a patient who is possibly in cardiac arrest
• The second action taken when finding a patient who is possibly in cardiac arrest
• The right action taken when finding a patient who is in cardiac arrest and not responding and no other person is available to help
• The specific practice during resuscitation of a patient with cardiac arrest with no pulse associated with improved survival
• The order of steps recommended by the current American Heart Association guidelines for adult CPR
• The right point to attempt a pulse check during CPR in adults
• The maximum duration of feeling for a pulse during CPR
• The proper hand placement/position in CPR
• The recommended rate for giving chest compressions in CPR
• The optimum compression–ventilation ratio for adult CPR when done by one person
• The recommended depth of chest compression in adults
• The recommended simple technique for clearing a patient's airway
• The device used when performing assisted ventilation during CPR in a hospital
• The way of assuring proper ventilation during CPR
• The need for rescue breathing in an adult rescue. Breathing is used for a patient who is unconscious but has a pulse
• The best way to open the airway in case of a suspected C-spine injury
• The recommended depth of chest compressions in children aged 1 to puberty
• The correct rate for rescue breathing in a child
• The optimum head position for the best ventilation of infants

The last item used to calculate the knowledge score was measured on a scale of 0–3, reflecting the critical characteristics of high-quality CPR. Finally, the score was normalized to a range between 0 and 100 to simplify understanding.

### 3.7. Statistical analysis

The statistical analysis was conducted using the IBM SPSS version 28 statistical package. Frequency distributions for selected variables were generated first. For quantitative variables known or assumed to be normally distributed, such as the calculated knowledge score, descriptive statistics such as the mean, standard deviation (SD), and standard error were used. The independent samples *t*-test was used to test the statistical significance of differences in means between two groups, while the ANOVA test was used to test the statistical significance of differences in means between more than two groups. An estimate was considered statistically significant if its *P*-value was less than an α level of significance of 0.05.

A multiple linear regression model was used to study the net and independent effect of a set of explanatory variables on a quantitative outcome (dependent) variable (the knowledge score).

### 3.7. Ethical considerations

Ethical approval was obtained from the PHCC Research Committee (PHCC RC). For skill assessment checklist completion, CBT members signed a consent form upon agreeing to participate. Adequate precautions were in place to assure voluntary participation of the targeted population. The research was executed in compliance with PHCC and MoPH human participant research ethics rules and regulations.

## 4. Results

A total of 569 physicians and nurses responded to the survey. Approximately three-quarters (72.6%) were females, and slightly less than a half (46.4%) had less than 5 years of clinical experience. Nurses constituted 78.1% of the sample. More than a half (62.7%) has been exposed to a real-life situation where they tried to revive a dying person with no pulse. Almost all (96%) expressed their confidence in CPR (know how to revive a dying person), and 96.8% formally received training on BLS. Only approximately a third (35%) had tried to revive a dying child with no pulse. Approximately half (48.7%) formally received a recent training (less than 1 year before) on basic life support. All personally believe that it is important to know how to revive a dying adult or child as part of their job (Supplementary File S1).

As shown in Table 1, the most frequently attended course was Immediate Life Support (ILS), with 71% of the sample having taken the course, followed by Basic Life Support (BLS), with 50.4%. The remaining courses, namely, ACLS, PLS, Advanced Life Support (ALS), and Advanced Pediatric Life Support (APLS), were less frequently attended (between 0.9% and 18%).

**Table 1:** Frequency distribution of the study sample by type of resuscitation training course attended in the last 3 years.

Total *N* = 569	*N*	%
Type of resuscitation training course attended in the last 3 years		
ILS	404	71.0
BLS	287	50.4
ACLS	106	18.6
PLS	91	16.0
ALS	45	7.9
APLS	5	0.9

In terms of measuring the attitude of respondents, more than 90% of the study sample expressed confidence and claimed knowledge and familiarity with CPR policy, guidelines, and alert procedures. However, two items showed an exceptionally lower proportion of confidence. Only 62.2% agreed that they were confident enough to insert a nasopharyngeal airway or laryngeal mask airway into a patient in an emergency, and 81% expressed confidence in their ability to defibrillate a patient if needed (Table 2).

**Table 2:** The relative frequency of positive attitude items.

Personal attitude (total *N* = 569)	*N*	%
Knowing how to assess the airway and breathing when confronted with a patient collapse	566	99.5
Confident enough to insert a nasopharyngeal airway or laryngeal mask airway to a patient in emergency situation	354	62.2
Being confident to defibrillate a patient if needed	461	81.0
Being familiar with the medications and their dosages used in resuscitation	521	91.6
Know how to alert code blue team of your health center in emergency	558	98.1
Know how to broadcast message and alert code blue team through the IP phone if faced with a clinical emergency	545	95.8
Know a code blue team exists in PHCC health centers	568	99.8
Know the latest CPR guidelines/algorithm	548	96.3
Read PHCC CPR guidelines/code blue policy	541	95.1
Feel a need for any additional resuscitation training in the future	503	88.4

The mean total knowledge score for the studied sample was 76.2%. A total of four studied characteristics had a statistically significant association with the magnitude of the mean knowledge score, including the duration of clinical experience in PHCC and overall, the professional affiliation, and the duration since formally receiving training on Basic Life Support/CPR. Being more experienced with a longer duration of clinical practice was associated with a higher mean knowledge score. Consultant physicians, specialists, and nurses had a higher mean knowledge score compared with senior consultants and residents. A shorter duration (<1 year) from the last formal training was also associated with a higher mean score. The remaining explanatory variables tested failed to show any obvious or statistically significant association with the mean knowledge score, including the health center region, having tried to revive/resuscitate a dying person/adult with no pulse, frequency of practicing resuscitation during the past 1 month, being confident that they know how to revive/resuscitate a dying person, having tried to revive/resuscitate a dying child with no pulse, duration since formally receiving training on Basic Life Support/CPR, and count of resuscitation courses attended in the last 3 years (Table 3).

**Table 3:** The mean total knowledge score by selected factors.

	Total knowledge score (/100)					
	Range	Mean	SD	SE	*N*	*P*
Gender						
Male	(24–96)	75.7	11.2	0.89	156	0.51 (NS)
Female	(20–96)	76.4	10.8	0.53	413	
Total	(20–96)	76.2	10.9	0.46	569	
Health center region						
Central	(20–96)	75.4	12.1	0.86	197	0.06 (NS)
Western	(52–96)	77.9	9.0	0.71	162	
Northern	(36–96)	75.6	10.9	0.75	210	
Years of clinical practice						
Less than 5 years	(24–96)	72.6	14.0	1.93	53	0.023
5–10 years	(40–96)	77.1	9.8	0.61	264	
More than ten years	(20–96)	76.1	11.0	0.69	252	
Years of clinical practice in PHCC						
Less than 5 years	(24–96)	75.6	10.7	0.59	329	0.041
5–10 years	(40–92)	78.2	9.5	0.79	145	
More than 10 years	(20–96)	75.3	12.9	1.32	95	
Profession						
Senior consultant	(20–80)	56.4	25.2	7.97	10	<0.001
Consultant	(48–92)	74.9	9.7	1.26	60	
Specialist	(56–92)	74.7	8.4	1.31	41	
Resident	(56–84)	67.3	9.3	3.78	6	
Head nurse	(68–84)	76.5	5.8	2.06	8	
Nurse supervisor	(60–88)	74.7	9.0	3.68	6	
Staff nurse	(36–96)	77.1	10.4	0.49	438	
Tried to revive/resuscitate a dying person/adult with no pulse						
No	(36–96)	75.1	11.8	0.81	212	0.07 (NS)
Yes	(20–96)	76.9	10.2	0.54	357	
Frequency of practicing resuscitation during the past month						
0	(36–96)	75.1	11.8	0.81	212	0.17 (NS)
1–2	(20–96)	76.8	10.3	0.55	344	
3–4	(56–88)	76.0	12.6	5.66	5	
5–6	(64–88)	81.5	8.3	2.92	8	
Know how to revive/resuscitate a dying person						
No	(48–92)	74.4	10.2	2.13	23	0.4 (NS)
Yes	(20–96)	76.3	10.9	0.47	546	
Tried to revive/resuscitate a dying child with no pulse						
No	(24–96)	76.1	10.8	0.56	370	0.7 (NS)
Yes	(20–96)	76.5	11.0	0.78	199	
Formally received training on Basic Life Support/CPR						
No	(24–88)	68.7	16.8	3.96	18	0.07 (NS)
Yes	(20–96)	76.5	10.5	0.45	551	
Duration since formally receiving training on Basic Life Support/CPR						
Less than 1 year	(28–96)	77.7	10.1	0.68	223	<0.001
Less than 5 years	(24–96)	76.0	10.2	0.61	277	
More than 5 years	(20–92)	72.2	14.3	1.72	69	
Count of resuscitation courses attended in the last 3 years						
Only one	(20–96)	75.4	11.9	0.67	312	0.08 (NS)
Two courses	(48–96)	76.7	9.8	0.77	164	
3–6 courses	(52–92)	78.2	8.4	0.87	93	

A multiple linear regression model was developed to evaluate the net and independent effect of a set of explanatory variables on the total knowledge score. The independent (explanatory) variables included in the model were gender, duration of clinical practice, practical experience (revive/resuscitate a dying person/adult with no pulse), frequency of practicing resuscitation during the past month, and frequency of attending resuscitation courses during the past 3 years. The model was statistically significant. Only three explanatory variables had no important or statistically significant association with the outcome, namely, gender, having a practical experience (revive/resuscitate a dying person/adult with no pulse), and the frequency of practicing resuscitation during the past month. Being a nurse is the most important factor in predicting a higher knowledge score. This health service provider role was associated with a statistically significant increase in the mean total knowledge score by a mean of 7% points compared physicians after adjusting for the remaining explanatory factors included in the model. Attending more resuscitation courses (three to six courses in the last 3 years) was associated with a statistically significant increase in the mean total knowledge score by a mean of 6.1% points compared with attending a single course only. In addition, a longer experience in clinical practice (5–10 and more than 10 years) was associated with a statistically significant increase in the mean total knowledge score by a mean of 3.3%–3.4% points compared with those with less than 5 years of experience (Table 4).

**Table 4:** Multiple linear regression model with total knowledge score as the dependent variable and a set of explanatory variables.

	Unstandardized coefficients	Standardized coefficients	*P*
(Constant)	72.1		<0.001
Male gender compared with female	0.1	0.003	0.94 (NS)
Duration of clinical practice (years)			
5–10 years of clinical practice compared with <5 years	3.4	0.156	0.035
>10 years of clinical practice compared with <5 years	3.3	0.152	0.04
Tried to revive/resuscitate a dying person/adult with no pulse	−0.9	−0.038	0.68 (NS)
Frequency of practicing resuscitation during the past month			
1–2 times compared with none	2	0.104	0.26 (NS)
3–4 times compared with none	4	0.21	
5–6 times compared with none	6	0.31	
Frequency of attending resuscitation courses during the last 3 years			
Attended two resuscitation courses in the last 3 years compared with only one	2.5	0.104	0.017
Attended 3–6 resuscitation courses in the last 3 years compared with only one	6.2	0.21	<0.001
Being a nurse compared with a being a physician	7.0	0.26	<0.001

*R*^2 ^= 0.08; *P* (model) < 0.001.

To measure CPR skills, a structured evaluation form was used to rate staff performance in practical skills, including the initial approach, compression, and ventilation techniques. The evaluation was based on the American Heart Association practical skills testing benchmark. The questionnaire was adapted and modified to suit local needs and resource availability (Supplementary File). A drill observation checklist was also utilized (Supplementary Material S2). Individual scores from the drill observation were assessed independently and in combination to produce an overall score for each participant. Due to the COVID-19 pandemic, data were obtained from only 14 health centers. The results of the CBT drill evaluation showed that it was conducted without any significant lapses. The performance of the CBT members from four health centers (more than a quarter of all health centers evaluated) was evaluated as satisfactory. The remaining 10 health centers (71.4% of all health centers evaluated) had some comments for staff skill improvement. These comments include the need for all team members to update their required training certificates, the need for further improvement in airway skills, the reaffirmation of roles and responsibilities of CBT staff members, the need to practice proper debriefing sessions, the stress on the fact that initial responders must be non-CBT members, the need to ensure high quality at all times with minimum interruption, the need to be familiar with the ACLS algorithm, the need to improve debriefing sessions, the absence of advanced airways which could facilitate uninterrupted chest compression and ventilation, failure of announcements to reach all staff, the need for discussion and further improvement in supraventricular tachycardia management, and finally, the failure of attendance by pharmacy and reception staff. Supplementary File S2 shows the percentage of evaluation items satisfactorily completed by a CBT member out of the total 14 teams that participated in the drill.

## 5. Discussion

Cardiovascular diseases require immediate effective intervention based on adequate knowledge and practical skills for resuscitation (14). The quality of this intervention is crucial for improving patient survival and quality of life (15, 16). Our study found that the level of CPR knowledge and skills displayed by the healthcare providers in PHCC HCs were satisfactory as most of them are well-trained and performed CPR in the past. Those practitioners were continuously monitored through CPR drills in their workplace and trained according to PHCC's Code Blue policy. In 2017, this policy was formulated to ensure that all PHCC health centers follow a standardized approach for handling and managing a person that appears to have suffered a cardiopulmonary/respiratory arrest or near arrest. The policy is intended to ensure that staff can respond appropriately and promptly to the patient in such situations. Similar studies conducted in other countries (14, 17–19) showed that CPR knowledge and skills among healthcare professionals, except those working in emergency settings, are comparatively less. This finding leads to the assumption that workload has a negative influence and results in a decline in CPR knowledge, given that it is not part of routine medical work (14), which calls for continuous training programs throughout the education cycle so that staff in training can be prepared to deal with these situations during training and beyond. Robak et al. (20) concluded that “learning through teaching” may improve self-confidence and provide in-depth knowledge by placing people in the situation of dealing with CPR regularly, whenever it is required (15, 21).

Since CPR is essential, healthcare providers should be competent in initiating and performing CPR irrespective of their training or work setting. Hospitals and healthcare centers should provide training to their staff (22, 23) since CPR proficiency among HCPs is significantly influenced by training (24) and is a major determinant of success (25). Routine training in CPR should be emphasized to acquire the necessary CPR knowledge (26). International recommendations suggest that HCPs should undergo a CPR course every 2 years (13, 25, 26). In many developing countries, standard resuscitation training is not routine (25, 27). In fact, it is often assumed that all HCPs can recognize and treat cardiopulmonary arrest (28).

The results of this study showed that the participants who had attended an introductory course had higher (though not statistically significant) knowledge of CPR. In addition, a shorter duration (<1 year) from the last formal training attended led to a significantly higher mean knowledge score. This finding is consistent with similar literature (29) that measured CPR skills and knowledge and showed that knowledge improved after attending the training. In this study, most participants know how to protect a collapsed patient. However, only 62.2% were confident enough to insert a nasopharyngeal airway or laryngeal mask airway into a patient in an emergency. This points out the importance of repeated education, training, and regular practice through CPR drills, which will help to maintain a high level of knowledge and CPR skills to keep the highest standard of service among healthcare workers. Again, these results are consistent with the previous study (21, 29, 30).

This study showed that healthcare workers attending more resuscitation courses (3–6 courses in the last 3 years) were associated with a significant increase in knowledge compared with those attending a single course only. In addition, a long experience in clinical practice (5–10 and more than 10 years) had a similar beneficial effect on CPR knowledge. These results are consistent with previous studies (15, 21, 25).

With regard to professional affiliation, this study showed that consultant and specialist physicians and nurses had higher CPR knowledge than senior consultants and residents. In general, nurses were more knowledgeable than physicians. Published literature showed that being a clinical nurse or a physician resulted in higher average ratings in the CPR test. The knowledge of CPR among nursing professionals (29–32) reflects their regular training (15, 31), and the same has been found for medical practitioners (21), whose ratings are higher after training. Other groups of professionals have lower response ratings, pointing to the fact that medical practitioners have more training and more exposure to medical emergencies (14). However, this must be assessed in various healthcare settings to determine if their staff are adequately trained and prepared to respond when needed (14, 33).

The CPR skill measurement and drill observation were assessed independently and in combination to produce an overall score for each participant. According to the collected data, the CBT drill was conducted without significant mistakes, and four HC staffs performed satisfactorily.

A limitation of this study was the inability to collect the data from all 27 HCs (only half of them were evaluated) due to the limitations of the COVID-19 pandemic. Despite this limitation, the results are informative because it helps us to understand the knowledge and skills of the healthcare providers in the public health centers of Qatar, which may serve as a basis for further studies on this subject. Furthermore, the results allow us to conclude that the knowledge of CPR was satisfactory, with a small percentage of the participants needing further improvement in airway management skills.

## 6. Conclusion

Considering that PHCC is the first step toward people's healthcare in Qatar, clinical staff should be certified and assessed regularly to ensure resuscitation knowledge and skills retention. The level of CPR knowledge and skills practice among healthcare providers in PHCC is deemed satisfactory as most providers reported having performed CPR in the past. More frequent training and continuously refreshing CPR certification (undergoing a recent training), especially for those healthcare workers with short work experience, are required for a high CPR knowledge.

## Data Availability

The original contributions presented in the study are included in the article/Supplementary Material, further inquiries can be directed to the corresponding author.
