# Supplementary material for: Knowledge, attitude, and proficiency of healthcare providers in cardiopulmonary resuscitation in a public primary healthcare setting in Qatar
Source: Front Cardiovasc Med. 2023 Jul 18;10:1207918. doi: 10.3389/fcvm.2023.1207918 (PMC10390828; doi:10.3389/fcvm.2023.1207918)
Supplement: Supplementary file 1 [file Datasheet1.pdf]

Supplementary Material 1.

Questionnaire 1

DEMOGRAPHICS

Please tick in the boxes provided

1. **Age:** ☐
2. **Gender:** Male ☐ Female ☐
3. **Profession:**

Doctors: Senior Consultant ☐

Consultant ☐

Specialist ☐

Resident ☐

Nurses: Head Nurse ☐

Nurse Supervisor ☐

Staff Nurse ☐

4. **Health Center Region**

Central ☐ Western ☐ Northern ☐

5. Years of clinical practice ________

Less than 5 years ☐ 5 to10 years ☐ More than 10 years ☐

6. Years of clinical practice in PHCC ________

Less than 5 years ☐ 5 to10 years ☐ More than 10 years ☐

Questionnaire 2

Baseline CPR training and CPR experience

1. Have you ever tried to revive/resuscitate a dying person/adult with no pulse?

Yes ☐ No ☐

2. If yes how many times in the past one month ☐
3. Do you know how to revive/resuscitate a dying person? Yes ☐ No ☐
4. If no, do you think it is important to know as part of your job Yes ☐ No ☐
5. Have you ever tried to revive/resuscitate a dying child with no pulse? Yes ☐ No ☐

If any of the 5 questions above is YES, please answer the questions below:

a. Have you duration since formally received training on Basic Life Support/CPR ? ☐

Yes No ☐

b. How long ago?

Less than 1 year ☐ Less than 5 years ☐ More than 5 years ☐

c. Do you know what a Defibrillator is? Yes ☐ No ☐

d. Have you ever been trained to use a Defibrillator? Yes ☐ No ☐

e. Do you have a Defibrillator in your Health Center? Yes ☐ No ☐

6. Resuscitation course have you done in the last 3 years

BLS ☐ ILS ☐ ALS ☐ ACLS ☐ PLS ☐ APLS ☐

Questionnaire 3

Service Experience

Circle the best answer/fill in the blanks

If you don't know the answer write IDN (I DON'T KNOW) besides the question.

If you can't understand the language/terminology inform the researcher for interpretation verbally.

1. What is the long form of "CPR" ____
2. You are alone and find a patient who is possibly in cardiac arrest. What will be your first response?
   a. Check for responsiveness
   b. Ensure scene safety
   c. Begin Chest Compressions
   d. Open the airway and give 2 breaths

3. You are certain of the above answer. What is your next step?
   a. Begin chest compressions
   b. Check for responsiveness
   c. Call the patient's doctor
   d. Provide rescue breathing

4. You are alone and find a patient who is in cardiac arrest and not responding, then what should you do?
   a. Certify death
   b. Shout for help
   c. Take blood samples
   d. Check the blood pressure

5. What part of resuscitation of a cardiac arrest patient with no pulse has been shown to improve survival?
   a. Early intubation
   b. Early defibrillation
   c. Giving adrenaline
   d. Placing a central line

6. The current American Heart Association guidelines for adult CPR recommend this order of steps:
   a. Chest compressions, Airway, Breathing
   b. Airway, Breathing, Check Pulse
   c. Airway, Breathing, Chest Compressions
   d. None of the above

7. When do you start CPR?
    a. There is no pulse and patient is not breathing
    b. There is a pulse and the patient is not breathing
    c. There is a pulse and the patient is unconscious
    d. All of above
8. Where should you attempt to perform a pulse check during CPR in adults?
    a. Brachial artery
    b. Ulnar artery
    c. Temporal artery
    d. Carotid artery
9. How long do you feel for a pulse during CPR?
    a. More than 10 minutes
    b. 1 hour
    c. Less than 10 seconds
    d. More than 10 seconds

10. Hand placement/position in CPR is:
    a. At the lower end of the chest and upper abdomen
    b. At the apex of the left part of the chest (On top of the heart)
    c. Center of the breast bone between nipples
    d. Upper part of the breast bone above the nipples

11. The recommended rate for giving chest compressions in CPR is at least _______ compressions a minute.

12. The correct compression-ventilation ratio for adult CPR when done by one person is _______compressions to ______ breaths.

13. What is the recommended depth of chest compression in adults?
    a. At least 1 inches (2.54cm)
    b. At least 1½ (3.56cm)
    c. At least 2 inches (5cm)
    d. At least 4 inches (10cm)

14. Healthcare providers should try to minimize interruptions in chest compressions to less than _______ seconds.
15. The critical characteristic/s of high-quality CPR includes which of the following?
    a. Starting chest compressions within 10 seconds of recognition of cardiac arrest
    b. Push hard and fast
    c. Minimize interruptions
    d. All of the above

16. A simple technique for clearing a patient's airway is:
    a. Head tilt, chin lift
    b. Push chin down, tilt head forward
    c. Lift chin up, turn head sideways
    d. Flex the neck
17. The following device is used when performing assisted ventilation during CPR in the hospital:
    a. A face shield
    b. Bag valve mask
    c. A face mask
    d. There is no preferred method

18. Correct ventilation during CPR is assured by:
    a. Visible abdominal rise
    b. Visible chest rise
    c. Coughing/gagging
    d. None of above
19. Rescue Breathing is used for a patient who is unconscious, but has a pulse. What is the correct rate for rescue breathing in an adult?
    a. 1 breath every 3 to 5 seconds
    b. 1 breath every 5 to 6 seconds
    c. 2 breaths every 3 to 5 seconds
    d. 2 breaths every 5 to 6 seconds

20. If you believe there is risk of a C-spine injury, the best way to open the airway is using
    a. Flexion technique
    b. Hyperextension technique
    c. Head tilt, chin lift technique
    d. Jaw thrust technique

21. The correct compression-ventilation ratio for children getting CPR when done by one person is _______compressions to ______ breaths.

22. What is the recommended depth of chest compressions in child aged 1 to puberty
    a. 1/3 to ½ of the depth of the chest
    b. At least ¼ of the depth of the chest
    c. 5cm or more
    d. 1cm or more
23. What is the correct rate for rescue breathing in a child?

    a. 1 breath every 3 to 5 seconds
    b. 1 breath every 5 to 6 seconds
    c. 2 breaths every 3 to 5 seconds
    d. 2 breaths every 5 to 6 seconds

24. For the best ventilation of infants, the head should be:
    a. Hyper extended

b. Neutral position
c. Flexed position
d. Turned to left lateral position

25. Do you know how to assess the airway and breathing if a patient collapse in front of you
    a. Yes
    b. No
26. Are you confident enough to insert a nasopharyngeal airway of laryngeal mask airway in to a patient in emergency situation
    a. Yes
    b. No

27. Are you confident to defibrillate a patient if needed
    a. Yes
    b. No

28. Are you familiar with the medications and their dosages used in resuscitation
    a. Yes
    b. No

29. Do you know how to alert code blue team of your Health Center in emergency
    a. Yes
    b. No

30. Do you know how to broadcast message and alert code blue team through the IP phone if faced with a clinical emergency
    a. Yes
    b. No

31. Do you know there is a code blue team exists in PHCC Health Centers
    a. Yes
    b. No

32. Do you know the latest CPR guidelines / algorithm
    a. Yes
    b. No

33. Have you read PHCC CPR guidelines/ code blue policy
    a. Yes
    b. No

34. Do you feel you need any additional resuscitation training in the future
    a. Yes
    b. No

Supplementary Material 2.

HEALTHCARE PROVIDER CPR/BLS SKILL DEMONSTRATION **CHECKLIST**

Health Center number ____________ Test date ______________________

During this phase we evaluate the physicians and nurses ability to initiate BLS and deliver a high quality CPR

Skill step	Critical criteria	Correct	Incorrect	Notes
1	**CHECK FOR RESPONSE**<br>- Check for response (inflict pain, tapping, hello)			
	- Time should be within 10 seconds			
2	**CALL FOR HELP**<br>- Call for help within 10 seconds			
	- Get defibrillator			
3	**PULSE CHECK**<br>- Check for pulse not more than 10 seconds			
	- Carotid pulse			
	- Between two heads of sternocleidomastoid muscle			
	- Finger tips use to check pulse			
4	**GIVE HIGH QUALITY CPR**<br>- Immediately not more than 10 seconds			
	- Correct position between the nipples, center of chest/lower half of the chest bone			
	- Two hands, one hand on top of the other using the heel of the first hand			
	- Extended elbow			
	- 30 compressions 10% lower and upper limit (record number)			

	- Adequate rate at least 100/min (count number of compression and divide with time on the video in the first cycle)			
	- Adequate depth at least 2inches in depth (75%) of compressions (There is a mark at the chin of the manikin)			
	Critical criteria	Correct	Incorrect	Notes
	- Allows complete chest recoil (75% of compressions)			
	- Minimize interruptions, hands off not more than 10 seconds			
	- Hand should be in contact with the chest			
5	**VENTILATION**<br>- Simple airway maneuvers (head tilt or chin lift)			
	- Correct technique of hand placement (fingers below the chin and above the bag)			
	- 2 breaths each over 1 second (within 10 seconds)			
	- Chest rise (All 2 breaths)			
	- Bag valve mask should cover the mouth and nose			
	- Compressions resumed immediately after breaths (within 10 seconds)			
	- From the end of the first compression to the start of the next compression time should be less than 10 seconds			

Supplementary file 1: Frequency distribution of the study sample by selected variables.

		N	%
	Gender		
	Male	156	27.4
	Female	413	72.6

		N	%
	Total	569	100.0
	Health CenterCenter Region		
	Central	197	34.6
	Western	162	28.5
	Northern	210	36.9
	Total	569	100.0
	Years of clinical practice		
	Less than 5 years	53	9.3
	5 -10 years	264	46.4
	More than 10 years	252	44.3
	Total	569	100.0
	Years of clinical practice in PHCC		
	Less than 5 years	329	57.8
	5 - 10 years	145	25.5
	More than 10 years	95	16.7
	Total	569	100.0
	Profession		
	Senior Consultant	10	1.8
	Consultant	60	10.5
	Specialist	41	7.2
	Resident	6	1.1
	Head Nurse	8	1.4
	Nurse Supervisor	6	1.1
	Staff Nurse	438	77.0
	Total	569	100.0
	Tried to revive/resuscitate a dying person/adult with no pulse		
	No	212	37.3
	Yes	357	62.7

		N	%
	Total	569	100.0
	Frequency of practicing resuscitation during the past one month		
	0	212	37.3
	1-2	344	60.5
	3-4	5	0.9
	5-6	8	1.4
	Total	569	100.0
	know how to revive/resuscitate a dying person.		
	No	23	4.0
	Yes	546	96.0
	Total	569	100.0
	Personally believe that it is important to know how to revive/ resuscitate a dying person as part of one's job		
	No	0	0.0
	Yes	569	100.0
	Total	569	100.0
	Tried to revive/resuscitate a dying child with no pulse		
	No	370	65.0
	Yes	199	35.0
	Total	569	100.0
	Duration since formally received training on Basic Life Support/CPR		
	No	18	3.2
	Yes	551	96.8
	Total	569	100.0
	Duration since formally received training on Basic Life Support/CPR		
	Less than 1 year	223	39.2

		N	%
	Less than 5 years	277	48.7
	More than 5 years	69	12.1
	Total	569	100.0

Supplementary file 2: Frequency distribution of selected assessment items in 12 clinics.

(N=12)	N	%
**Basic Requirements related objectives**		
The Health Center have identified Code Blue team as per the policy	12	100.0
The CODE BLUE Team leader, TM1and TM2 have valid BLS, ACLS and PLS- PALS-AHA Certificates	11	91.7
The CODE BLUE TM3, TM4 and TM5 have valid BLS/ ACLS- AHA Certificates	11	91.7
The Health Center Manger Office /Head Nurse keep a mock drill book to record mock CODE BLUE drills (Code Blue folder is completed	12	100.0
The CODE BLUE drill conduct in Monthly basis	12	100.0
The daily and Monthly E-Cart checklist was completed as per E-Cart policy	12	100.0
The defibrillators testing was done every shift print out available on daily basis	11	91.7
All equipment /Cardiac Board, medications available/functioning for both adult and pediatric emergencies	12	100.0
**Basic Cardiac Resuscitation Simulation-General preparation**		
All the CODE BLUE team members arrived within an average of 3 minutes of activation the CODE BLUE	11	91.7
The CODE BLUE team leader was identified during the incident	12	100.0
The CODE BLUE team leader distributed the task among the team members as per the policy	12	100.0
**The CPR for adults initiated in following sequence as per policy. The first responder roles/HCP (CODE BLUE activator)**		
Checked scene safety	10	83.3
Determined of responsiveness of the victim.	12	100.0
Called for help.	12	100.0
Positioned the victim.	9	75.0
Determined absence of breathing and 6. check pulse (simultaneously) within 10 seconds	11	91.7
Called for help and activate CODE BLUE response	12	100.0
Initiated CPR	12	100.0
If no Breathing or no normal breathing and no Pulse. ACTIVATE CODE BLUE. direct the initial PHCC personnel to call EMS @ 999	11	91.7
Commenced initial High Quality CPR for adult, started by chest compressions as per C-A-B process.	12	100.0
Cardiac Board available and used?	8	66.7
Gave thirty (30) chest compressions.	12	100.0

(N=12)	N	%
Maintained compression rate at 100-120/min.	12	100.0
Maintained compression depth for adults should be 2 Inches (5cm) no more than 2.4 inches (6 cm)	12	100.0
Maintained compression an open airway and give two (2) rescue breaths using the proper Bag-Valve –Mask device .	11	91.7
Continued with 30:2 sequences.	11	91.7
Stopped CPR if patients show signs of life/ breathing, pulse, speaking, eye opening, etc.)	12	100.0
Stopped CPR if EMS team arrived to the scene.	12	100.0
**Code Blue Team Responsibilities-TM (The CODE BLUE Team Leader)**		
The CODE BLUE Team Leader is a Physician who is (ACLS from AHA) Certified	12	100.0
The CODE BLUE Team Leader Initiated and performs immediate Resuscitation	11	91.7
The CODE BLUE Team Leader received report of situation from personnel who have been attending the resuscitation event from the beginning.	12	100.0
The CODE BLUE Team Leader directed and supervises the activities and performance of the CODE BLUE team.	12	100.0
The CODE BLUE Team Leader ensured High-Quality CPR all times	10	83.3
The CODE BLUE Team Leader verified that Crash cart containing oxygen cylinder, Bag Mask Device, AED, suction is available at the scene.	11	91.7
The CODE BLUE Team Leader assigned a nurse to ensure Emergency Medical Service is on their way.	11	91.7
The CODE BLUE Team Leader recognized of the current ECG rhythm and Apply ACLS 2015 AHA Guidelines	10	83.3
The CODE BLUE Team Leader initiated the Resuscitation Medications administration as per ACLS- AHA latest protocols.	12	100.0
The CODE BLUE Team Leader ensured that monitor/ defibrillator is attached to patient and intravenous line is established / available;	12	100.0
The CODE BLUE Team Leader safely recognized the need for defibrillation, temporary pacing, interpretation of laboratory results, order ventilation, with Bag Mask device, endotracheal intubation, etc.)	9	75.0
The CODE BLUE Team Leader signed the CPR Record for medications given	9	75.0
The CODE BLUE Team Leader facilitated post Code Blue debriefing discussions	8	66.7
**Code Blue Team Responsibilities-TM1 (Physician who is ACLS certified from AHA)**		
TM1 (Physician who is ACLS certified from AHA) performed the D/ C shock delivery if ordered by the CODE BLUE Team Leader	11	91.7
TM1 (Physician who is ACLS certified from AHA) ensured clearing the victim before Defibrillator /AED, analyzing the rhythm and delivering the D/C Shock	12	100.0
TM1 (Physician who is ACLS certified from AHA) attached the AED and Defibrillates	11	91.7

(N=12)	N	%
TM1 (Physician who is ACLS certified from AHA) directed the resuscitation when the resuscitation team leader is not available	9	75.0
Code Blue Team Responsibilities-TM2 (physician who is ACLS certified from AHA)		
TM2 (physician who is ACLS certified from AHA) assisted in patient Airway Management	12	100.0
TM2 (physician who is ACLS certified from AHA) ventilated with a Bag Mask Device with the assistance of oropharyngeal/Nasopharyngeal Airways.	12	100.0
TM2 (physician who is ACLS certified from AHA) ensured optimal SPO2 level throughout the CODE BLUE by delivery of rescue breaths and oxygen supply.	8	66.7
TM2 (physician who is ACLS certified from AHA) attached the SPO2 sensor to the Victim	8	66.7
TM2 (physician who is ACLS certified from AHA) considered insertion of the Advanced Airway when ordered by the CODE BLUE Team Leader.	11	91.7
**Code Blue Team Responsibilities-TM3 (a Staff Nurse who is BLS-AHA certified from ACLS)**		
TM3 (Staff Nurse who is BLS-AHA certified from ACLS) started and assisted with external cardiac compression after ensured the placement of the CPR backboard and considered TM2 as chest compression relievers.	12	100.0
TM3 (Staff Nurse who is BLS-AHA certified from ACLS) immediately resumed the Chest Compression after the Shock Delivery	11	91.7
TM3 (Staff Nurse who is BLS-AHA certified from ACLS) assisted with Post Resuscitation Care.	12	100.0
**Code Blue Team Responsibilities-TM4 (a Staff Nurse who is BLS, AHA/ACLS certified from AHA)**		
TM4 (Staff Nurse who is BLS, AHA/ACLS certified from AHA) initiated IV Access by inserting the largest gauge cannula if possible.	12	100.0
TM4 (Staff Nurse who is BLS, AHA/ACLS certified from AHA) collected necessary blood samples.	12	100.0
TM4 (Staff Nurse who is BLS, AHA/ACLS certified from AHA) started an initial intravenous fluid as ordered only by the CODE BLUE Team Leader.	12	100.0
TM4 (Staff Nurse who is BLS, AHA/ACLS certified from AHA) administered emergency drugs as per CODE BLUE Team Leader orders only	12	100.0
**Code Blue Team Responsibilities-TM5 (a Staff Nurse who is BLS-AHA/ ACLS certified from AHA)**		
TM5 (Staff Nurse who is BLS-AHA/ ACLS certified from AHA) responded immediately to the code call.	12	100.0
TM5 (Staff Nurse who is BLS-AHA/ ACLS certified from AHA) ensured EMS is on their way	12	100.0
TM5 (Staff Nurse who is BLS-AHA/ ACLS certified from AHA) recorded and take responsibility for documentation	12	100.0

(N=12)	N	%
TM5 (Staff Nurse who is BLS-AHA/ ACLS certified from AHA) kept track of the time and frequency of resuscitation cycles	12	100.0
TM5 (Staff Nurse who is BLS-AHA/ ACLS certified from AHA) assisted the Head Nurse/ Charge Nurse /Designee to complete the OVA on DATIX.	8	66.7
**Code Blue Team Responsibilities-The Head and /or in Charge Nurse**		
The Head and /or in Charge Nurse completed CPR and critique record with ongoing information.	10	83.3
The Head and /or in Charge Nurse followed resuscitation event, review CPR and evaluate the record	8	66.7
The Head and /or in Charge Nurse verified the availability of Crash cart	10	83.3
The Head and /or in Charge Nurse assisted in two rescuers CPR if needed or if insufficient personnel present.	10	83.3
The Head and /or in Charge Nurse arranged to supply additional urgently required consumables and equipment during the code	9	75.0
**Code Blue Team Responsibilities-Pharmacist**		
The Pharmacist responded immediately to the code call	10	83.3
The Pharmacist assisted in preparing drugs as requested.	9	75.0
The Pharmacist maintained and provides back-up drug box.	8	66.7
The Pharmacist ensured replacement of crash cart drugs.	8	66.7
The Pharmacist ensured Resuscitation Medications replenishment during the Code Blue	7	58.3
**Code Blue Team Responsibilities-security/Hayyak personnel**		
The security/Hayyak personnel dealt with family members with Hayyak representative if available	7	58.3
The security/Hayyak personnel dealt assisted in crowd control and ensuring that the patient family is notified of the patient condition by the Doctor in charge	6	50.0
